# Material research for environmental sustainability in Thailand: current trends

**DOI:** 10.1088/1468-6996/16/3/034601

**Published:** 2015-05-05

**Authors:** Panadda Niranatlumpong, Nudjarin Ramangul, Pongsak Dulyaprapan, Siriluck Nivitchanyong, Werasak Udomkitdecha

**Affiliations:** National Metals and Materials Technology Center, Pathumthani, Thailand

**Keywords:** Thailand, material research, environmental policy

## Abstract

This article covers recent developments of material research in Thailand with a focus on environmental sustainability. Data on Thailand’s consumption and economic growth are briefly discussed to present a relevant snapshot of its economy. A selection of research work is classified into three topics, namely, (a) resource utilization, (b) material engineering and manufacturing, and (c) life cycle efficiency. Material technologies have been developed and implemented to reduce the consumption of materials, energy, and other valuable resources, thus reducing the burden we place on our ecological system. At the same time, product life cycle study allows us to understand the extent of the environmental impact we impart to our planet.

## 1. Introduction

Material science and engineering was one of the key success factors of the industrial revolution in the late eighteenth to early nineteenth century, which began in Great Britain [1]. The revolution brought about a better living standard for the people at large but at the same time put more strain on our environment [2, 3]. Coal-fired steam engines to drive transport and machinery, together with iron production and mechanical and thermal metal processing, led the way in both respects [4]. Since that era our demand for manufactured products has not subsided. This demand is driven by population growth as well as advancements in technology, material science included. With continued global growth, demand will continue to grow. With our fast-moving high-tech lifestyle, demand continues to grow. In the last 20 years, the world population has increased by 26% but extraction of natural resources and food production has increased by more than 40% [5]. All of this bodes poorly for our already deteriorating environment. As members of the global society and as material technologists, it is our duty to rise to this challenge and to protect our planet’s environment.

Environmental challenges faced by Thailand in many ways follow those of the global society: climate change, water crisis, food crisis, and energy stability. The complexity of their interdependence makes it impossible to consider them separately. We can tackle various issues at the national level, beginning with encouraging public awareness, establishing sustainable management systems, maintaining up-to-date product life cycle databases, etc. These are part of the framework required to allow a successful implementation of ‘environmentally less hostile’ technologies.

**Figure 1.**
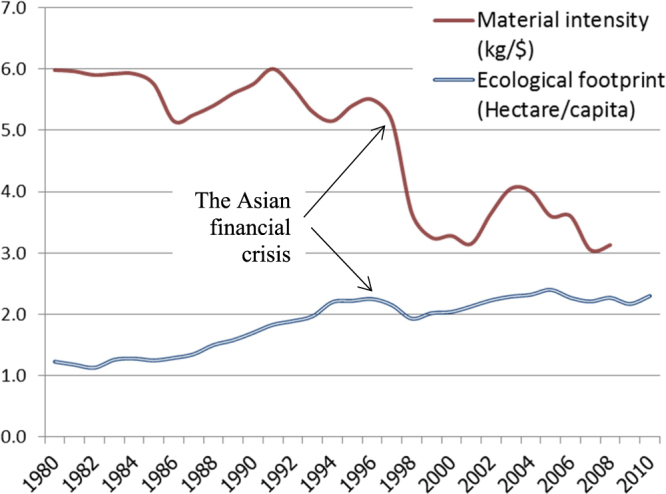
Material intensity and ecological footprint among Asian countries [6, 36].

In terms of technological advancement, Thailand has focused its material research and development over the last 40 years largely on production efficiency. This includes resource utilization and energy expenditure. Thailand’s material intensity (material usage per GDP) has improved significantly (see figure 1). This reduction in material intensity values suggests that from 1980 to 2008 Thailand showed significant improvement in making more efficient use of materials. However, when compared with other Asian countries in 2008, Thailand’s material intensity was still much higher than that of Japan and the Republic of Korea [6]. The Asian financial crisis which started in 1997 is responsible for the marked decrease in material intensity values for many years that followed. As the situation recovered in 2003, material intensity increased with economic growth to approximately 4 kg/USD, whereas it continuously decreased to approximately 3 kg/USD in 2008.

The impact of Thai industries on the environment has been recognized by the government and has been included in the National Economic and Social Development plan since 2002, and it was included in the first National Science Technology and Innovation Policy and Planning document issued by the Ministry of Science and Technology of Thailand in 2012 [7].

The National Metal and Materials Technology Center (MTEC), as a member of the National Science and Technology Development Agency (NSTDA), is a government-sponsored institute. MTEC plays a central role in material research and development in Thailand. Together with our partner institutes, universities, and funding agencies, we strive to reach the national goal of eco-efficiency and environmental sustainability of economic growth.

Under the theme of environmental protection, research at MTEC is focused on three main areas: (a) resource utilization, (b) material engineering and manufacturing, and (c) life cycle efficiency.

## 2. Resource utilization

**Figure 2.**
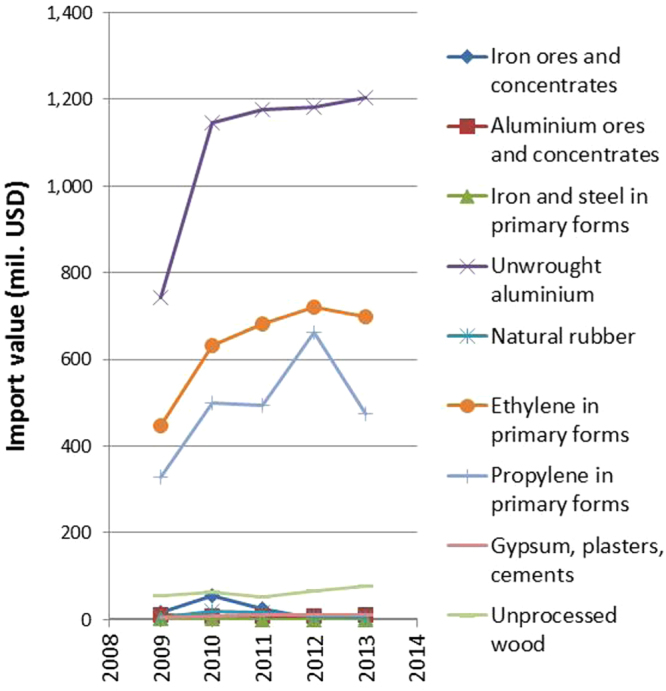
Thailand’s import data of primary materials.

Thailand does not produce many raw materials in large quantity apart from natural rubber, gypsum, and cement. The import figures show an increasing trend in imports of raw materials of several types, including aluminum and ethylene (see figure 2). Our research and development concentrate on using these resources to their full potential. Some examples are given hereafter.

### 2.1. Powder metallurgy

Powder metallurgy (PM) is a good example of a processing technology where material resources can be used efficiently. In terms of resource utilization, PM processes produce parts with precise tolerances; hence machining steps and waste are greatly reduced. For high-volume production of small, high-precision components, PM can be a more economical and environmentally friendly process route when compared with other processing routes such as material removal or precision casting.

One advantage of the PM process over the traditional casting process is the wider range of microstructures that can be obtained. Powder preparation and post treatment can be used to manipulate structures, for example, the preparation of powder containing Cu_6_Sn_5_ intermetallic or CuO nanoparticles to obtain products with unique properties [8, 9]. The development of compacting, sintering, and heat treatment techniques for new materials is also required to support the growth of PM in Thai industries [10–12].

**Figure 3.**
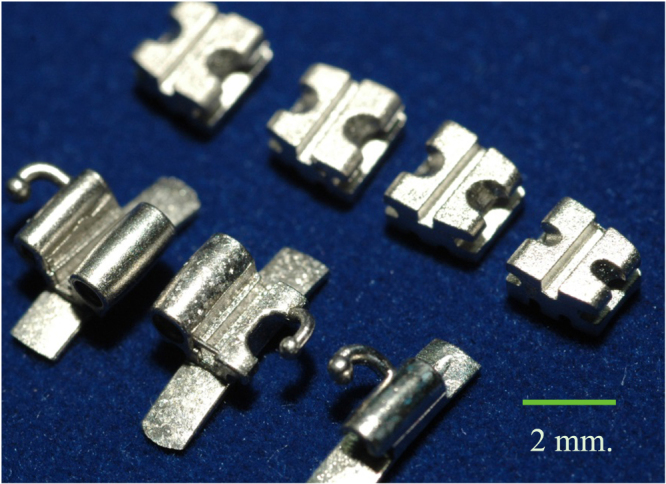
Dental brackets produced via MIM.

Metal injection molding (MIM) as a branch of PM finds applications in the electronics and jewelry industries in Thailand. In MIM, a mixture of fine metal powder and binder is processed using injection molding. Understanding the rheology of the feedstock is vital for achieving a high-quality finished product. MIM of metals and alloys of high value such as Ni, Ti, and rare earth metals gains an economic edge due to the near-zero-waste concept. These elements are often scarce, and mining leads to disturbance of the natural landscape. Furthermore, use of indirect resources such as machine tools, chemicals, water, and energy can also be reduced. Work has been carried out to gain insight into MIM processes and to improve the processing parameters which will lead to a wider range of applications (see figure 3) [13–15].

### 2.2. Rubber processing

Thailand produces its own latex and natural rubber and exports them in large quantities. To date, rubber plantations cover more than 20 million hectares, and their area is continuously growing in response to the global demand for natural rubber. Rubber is therefore one of the strategic research targets identified for the country. Much effort has been invested in increasing plantation yields, not only in terms of cultivars but also in terms of post-harvest processing. Increasing farm yield will eventually reduce the rate of deforestation. MTEC’s material research in post-harvesting includes work on prolonging the life and preserving the quality of latex, a high-efficiency coagulation process, and an eco-friendly coupling agent for rubber compounds [16–18].

### 2.3. Cement fillers and alternatives

Cement production is one of the largest producers of greenhouse gas among industrial sectors. It is also one of the fastest growing industrial sectors in Thailand. CO_2_ is released during the heating of calcium carbonate. The cement industry is responsible for approximately 5% of global industrial CO_2_ emissions [19]. To reduce the impact of the industry, we need to slow the demand for cement. Work has been done on replacing some of the cement with fillers such as plant fibers, fly ash, and rice husk ash, with the added benefit that these fillers are often unwanted byproducts of other industries [20, 21]. Parallel work is also under way on alkali-activated aluminosilicate-based geopolymer as a replacement for cement in construction. This material not only has the potential to replace conventional cement but can also make use of waste from coal-fired power plants [22].

### 2.4. Natural materials

**Figure 4.**
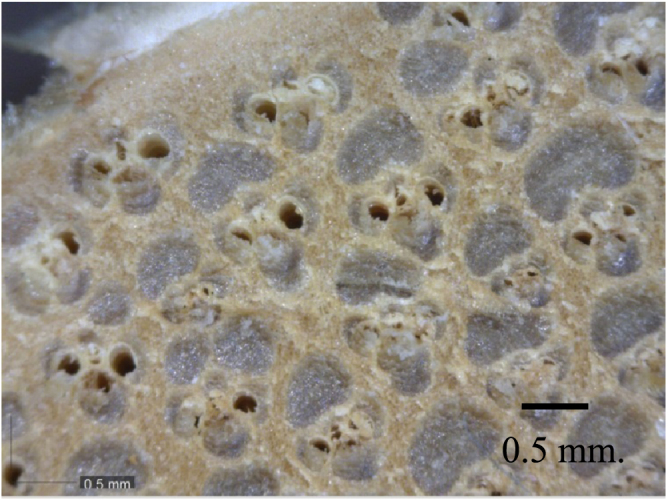
Cross-section of bamboo grains.

Wood remains a popular choice as a construction and household material. In furnishing, wood is preferred due to its conventionality, comfort, strength, and, in many cases, energy conservation. Bamboo is a fast-growing alternative to tree woods. A bamboo forest can be harvested after just three years of plantation. Well-managed bamboo farms trap a relatively high quantity of CO_2_ per hectare of managed forest area [23]. Furthermore, in Asian culture bamboo shoots are also valued as a food source. The challenges in replacing traditional tree woods with bamboo involve chemical treatment against mold, pests, and discoloration as well as joining. Figure 4 reveals the porosity of dry bamboo, which encourages the growth of biodegrading organisms. This area of research has recently been introduced at MTEC. We aim to replace some traditional woods with low-cost, low-life-cycle-cost bamboo.

## 3. Materials engineering and manufacturing

Thailand as a processing hub for automobile and electronics parts for the Southeast Asia region supports many research projects in materials engineering and processing. This research aims to achieve at least one of the following objectives: produce high-quality parts with longer life, thus reducing material consumption; minimize waste; or minimize energy consumption. Some of our projects are outlined in the following paragraphs.

### 3.1. Aluminum processing

Aluminum requires a large amount of energy in its purification process. This metal, however, is 100% recyclable. Recycling of aluminum generates a significant energy saving over the production of primary aluminum and helps to reduce the demand for landfills [24]. Thailand produced approximately 2.5 million cars in 2012 [25, 26]. On average each car uses approximately 70–100 kg of aluminum [27]. Therefore, as much as 200 000 tons of aluminum in Thailand ended up in automotive parts. This is accountable for approximately 70% of the total use of aluminum in Thailand. The common defect rate of 5–7% found on the shop floors of cast houses and with other uses of aluminum brought the total consumption of aluminum in 2012 to 300 000 tons. Continuous process improvement and defect reduction programs along with process optimization are strongly advocated by automotive manufacturers to eliminate waste and minimize the consumption of energy. Processes such as squeeze casting and semi-solid casting of aluminum alloys provide alternatives for Thai industry to achieve high-quality products with lower waste and lower energy consumption [28]. Consultation on material control and process optimization is offered to industries to encourage the use of secondary ingot, returns, and suitable scrap [29]. This is also one of the areas where successful implementation is dependent on the public framework regarding the waste management system (collection, sorting, and recycling of scrap) being in place along with major players in the automobile field supporting the national policy.

### 3.2. Polymer engineering

**Figure 5.**
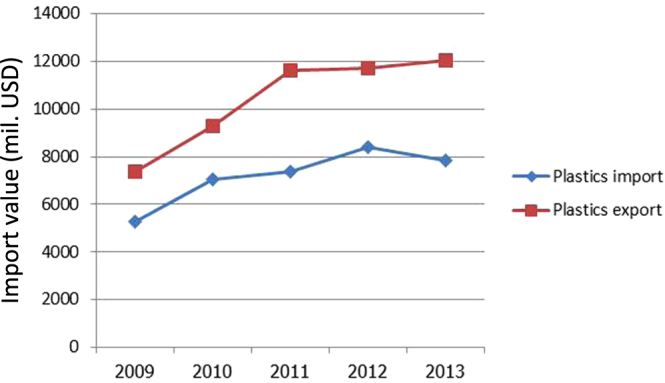
Thailand’s import/export data of polymers in millions of US dollars.

Polymer engineering is another major contributor to the country’s GDP. Figure 5 shows the growth of the import/export figures for polymer raw materials and polymer products. Polymers are featured prominently in our lives, from packaging to electronics. Research and development in polymer engineering have strong potential to create significant impacts not only on the economy but also on society in general and the environment. MTEC’s work in this field is focused on studies of polymer synthesis as well as reinforced polymers and their processing [30–32]. The information and technology gained from research and development with respect to process improvement and energy efficiency are made available to industries via seminars, training programs, and local publications.

### 3.3. Surface engineering

Surface engineering enhances the performance of a component. It enables the use of cheaper, more abundant materials in demanding applications. Surface engineering can also prolong the lifetime of products, thus reducing the use of natural resources in the long term. Surface techniques such as carburization, electroplating, thermal spraying, and physical vapor deposition serve a wide range of industries such as jewelry, agriculture, electronics, automobiles, and power generation. Our research priority has been on wear-resistant and corrosion-resistant applications to increase the reliability of components [33, 34]. The application of wear-resistant coatings and solid lubricant coatings has been successfully implemented in Thai industry. In addition to reducing the use of materials, a suitable coating application can help reduce the amount of energy required for operation due to reduction in friction [35].

## 4. Life cycle efficiency

The human population currently consumes resources at a higher rate than the world can produce them. Biocapacity is a term that describes the capacity of our ecosystems to produce useful biological materials and to absorb waste materials using current technologies. Ecological footprint is a measure of the area of biologically productive land and water an individual requires to produce all the resources he/she consumes and to absorb the waste generated.

Figure 1 shows an alarming trend in Thailand wherein the ecological footprint is continuously growing whereas Thailand’s biocapacity remains unchanged at just under 1.2 hectares per capita [36]. A major change occurred around 1985, when Thailand underwent a gradual transition from an agrarian to an industrialized economy. The industrial boom in the region caused the ecological footprint to increase to almost double that in the pre-industrialization era. The Asian financial crisis that started in 1997 set back the country’s industrial activity significantly, which is evident as a drop in the ecological footprint. Since then, as the economy has recovered, the figure has also climbed back up. Even though the material intensity values in figure 1 show a significant improvement in 2007 and 2008, the ecological footprint has maintained a relatively steady value at around 2.2–2.3 hectares per capita.

It is thus important that the use of resources be justified in every aspect of our lives if we are to reduce the gap between biocapacity and ecological footprint. In manufacturing, for instance, we need to assess every activity required to make a product, from digging up ore and extraction of petroleum compounds all the way to manufacturing and design, logistics, product uses, and end-of-life management.

### 4.1. Life cycle analysis

Life cycle analysis (LCA) assesses the ecological impact of a product from the beginning of production to the end of its life. It is often a matter of debate where a product’s life ends: up to the point when its useful life is finished, when it is upcycled into a new product, or when its waste is decomposed and absorbed back into the Earth? Assumptions need to be made to suit the context and the purpose for which the analysis was designed. A well-constructed LCA together with a comprehensive database allows well-informed decision making in various respects. From the manufacturing side, LCA can point out the hot spot in a production line. From the consumer side, it reveals any hidden cost, thus allowing the consumer to make an informed choice. At the national level, it can point out impending risk to the nation and allows some time to prepare a countermeasure. Work on LCA in Thailand focuses primarily on creating a practical database for materials processing and on implementation of LCA concepts in our industries [37–40].

### 4.2. Eco-design

A design process involves consideration of the cost and functionality of a product. An eco-design process puts emphasis on environmental impact, sometimes to the point where the reduction of impact becomes the goal of the activity. The task of an eco-designer is therefore to create an alternative product with the same function but with the lowest possible ecological cost. Research institutes, the education sector, and the private sector in Thailand are working together to promote the eco-design concept through training, consultations, and public events.

The successful development of eco-products such as electrical appliances and packaging requires a manufacturer’s full involvement in the start-up of these projects [41]. National policy also plays a vital role in establishing interest in such a scheme. Thailand’s eco-car initiative is one such example. In 2007, Thailand’s Board of Investment (BOI) launched its first phase of the eco-car program, where incentives were offered for integrated car assembly and key parts manufacturing projects. Excise tax incentives were also offered by the Finance Ministry of Thailand to foster domestic demand. This program succeeded in involving key manufacturers and attracting foreign investment to facilitate a new product. The second phase of the program, involving nine automakers, was approved in 2014. These automakers are required to commence production of new eco-cars by 2019.

### 4.3. Waste management

**Figure 6.**
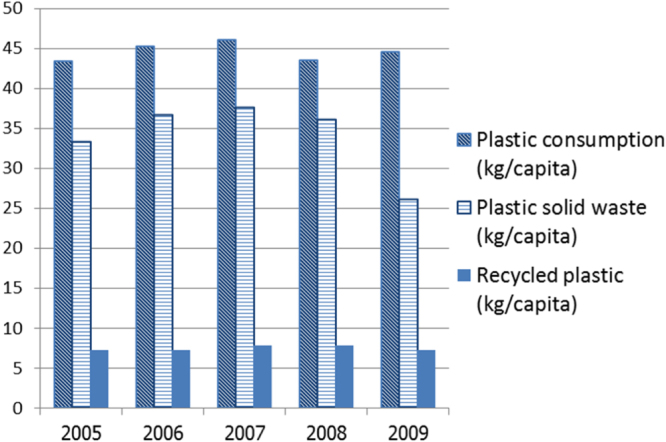
Plastic consumption per capita in comparison with the volumes of plastic solid waste (PSW) and recycled plastic.

Paper and packaging materials are the largest contributors to household waste after food waste. Paper is currently routinely recycled, but only some plastics can be recycled economically (see figure 6). Research has been carried out to study the recyclability of engineering polymers [42]. Within the past 10 years, the rate of recycling of plastics per capita in Thailand has been approximately 16–17% [43]. The remainder of the plastic waste is deposited in landfills. Biodegradable packaging is thus under development with the aim of replacing slow-disintegrating polymers such as polystyrene packaging [44, 45]. To facilitate matters for the packaging industry and to stimulate public awareness, a bioplastic testing laboratory has also been established in Thailand.

## 5. Conclusion

The economy in the Southeast Asia region is expected to continue to grow, which will place more burden on our environment. Thailand has demonstrated success in reducing the waste and material intensity in processing, which is helping to improve resource expenditures. However, more data are needed to be able to show the full picture. Therefore, there is a clear need for an improved LCA database and continued research and development regarding the reduction of resource expenditures and the reduction of waste in materials processing. Such a refinement of current technologies is necessary, but it can have only limited scope. More drastic improvement requires a significant change in processing, and this highlights the need for research into alternative materials with lower ecological footprints, extension of a product’s useful life, and recycling technology to further reduce the impact of industry on the environment.

Environmental issues are already featured predominantly in Thailand’s national policy, which sets the industrial development trend toward more energy-efficient and resource-efficient practices. Because a large proportion of research projects in Thailand is government funded, such a policy is a major driving force for scientists and engineers to work together in finding sustainable solutions for the current human-related issues affecting our environment.
